# Emotion Regulation Modulates Affective Responses Without Altering Memory Traces: A Study of Negative Social Feedback from Acquaintances

**DOI:** 10.3390/bs15091294

**Published:** 2025-09-22

**Authors:** Peng Liu, Xin Cheng, Mengyao Fan, Zhichao Huang, Chao Zhang

**Affiliations:** School of Psychology, Shanxi Normal University, Taiyuan 030031, China

**Keywords:** negative social feedback, cognitive reappraisal, distraction, negative memory forgetting, depression

## Abstract

Negative social feedback can cause social pain and may damage physical and mental health. In particular, negative social feedback from acquaintances deeply activates the social pain brain network between the dorsal anterior cingulate cortex (dACC), the anterior insula, and the amygdala, inducing stronger emotional responses and memories. This study used a social appraisal paradigm to investigate the potential benefits of emotional regulation in the face of negative social feedback from acquaintances, as measured by emotional responses and memories. The results showed that negative social feedback sent by acquaintances induced stronger emotional experiences and deeper negative memories than those sent by strangers. Cognitive reappraisal and distraction could reduce the negative emotions induced by negative social feedback sent by acquaintances; however, they did not affect the forgetting of memories of negative social feedback. Further analyses revealed that the emotion regulation strategy was more effective in alleviating negative emotions in the group with self-reported low-depressive symptoms compared to the group with self-reported high-depressive symptoms. Thus, the study suggests that the effectiveness of emotional regulation strategies varies across different relational contexts.

## 1. Introduction

### 1.1. Social Feedback

Social feedback conveys important interpersonal information, including others’ evaluations of one’s appearance, personality traits, behavior, and willingness to interact (Ruff & Fehr, 2014). Social feedback is critical to social interactions (Brudner et al., 2023), and an impaired ability to appropriately adjust behavior in response to social feedback can lead to poor interpersonal relationships and unpleasant social encounters (Frey et al., 2021). Such negative social stress—emotions that are not effectively detoxified and persist over time—can lead to individuals being at risk of various psychiatric disorders (Cludius et al., 2020). Notably, compared to evaluations from strangers, social feedback from intimate people often elicits stronger and more persistent emotional responses due to its greater social salience (Xie et al., 2022), making research on social feedback in intimate relationships more relevant to real-life situations. Therefore, effectively regulating negative emotions triggered by negative social feedback from acquaintances and facilitating the fading of negative memories plays an important protective role in dealing with intimate relationships and maintaining individual mental health.

### 1.2. The Role of Emotion Regulation

Emotional regulation is the process by which individuals influence the occurrence, experience, and expression of emotions (Gross, 1998). When faced with negative life events, people often actively employ regulatory strategies to adjust their emotions and cognition. Gross described two main categories of emotional regulation strategies: proactive and reactive. Proactive emotional regulation occurs before the tendency toward an emotional response is fully activated, whereas reactive emotional regulation occurs after an emotional situation has triggered the tendency to respond (Gross, 1998; Gross & John, 2003). Cognitive reappraisal is a proactive emotion regulation strategy that requires individuals to understand potential emotion-inducing situations in a different (e.g., non-emotional) way (Gross & John, 2003). In particular, the role of cognitive reappraisal in reducing emotional responses has been the focus of many studies. This strategy typically relies on the prefrontal cortex (e.g., the ventrolateral prefrontal cortex [vlPFC]) regulating emotional brain regions (e.g., the amygdala) (Ochsner et al., 2004). In interactions with familiar individuals, securely attached people can still effectively activate their prefrontal regulatory network (Mikulincer & Shaver, 2007), thus, reducing emotional intensity by reinterpreting feedback motivation (e.g., “He’s doing this for my own good”). fMRI evidence further supports this view, as activation of the vlPFC during reappraisal of a partner’s criticism still predicts reduced emotional ratings (Hooker et al., 2010). In addition to cognitive reappraisal, distraction is a commonly used and effective emotion regulation strategy in daily life (Xie et al., 2023; Li et al., 2022; Nasso et al., 2020). During distraction, the dorsal anterior cingulate cortex’s (dACC’s) activation toward the amygdala is reduced (McRae et al., 2010). This, in turn, decreases the dACC’s sensitivity to social rejection signals (Eisenberger et al., 2003) and reduces ruminative activity in the default mode network (Andrews-Hanna et al., 2010). Research indicates that distraction can alleviate the distress caused by negative feedback in intimate relationships (Woo et al., 2014), demonstrating its unique emotional regulation advantages. Furthermore, the use of cognitive reappraisal and distraction strategies in daily life can predict an increase in positive emotions (Boemo et al., 2022). Based on the above research, this study will focus primarily on cognitive reappraisal and distraction as two adaptive emotional regulation strategies. However, in addition to reducing negative emotional responses, this study will also ask the following question, does cognitive reappraisal and distraction also help people forget memories related to negative social feedback?

Previous studies have preliminarily explored how emotional regulation strategies influence emotional memory. Some scholars argue that emotional regulation itself consumes a certain amount of psychological resources. When individuals engage in energy-consuming regulatory behaviors, the resources available for cognitive activities such as information encoding are reduced, thereby impairing accompanying cognitive processing (Baumeister et al., 1998; DePaulo et al., 1992). However, some researchers (Gross, 1999) argue that different emotion regulation strategies have distinct mechanisms of action. For example, cognitive reappraisal occurs before the emotion is generated, does not require sustained self-regulation, and does not consume additional cognitive resources to manage emotional information. Thus, it does not impair the quality of memory task performance. Current research remains divided on the effects of cognitive reappraisal on emotional memory (Davis & Levine, 2013; Egloff et al., 2006; Hayes et al., 2010; Richards et al., 2003; Richards & Gross, 2000; Sheppes & Meiran, 2008; Xie et al., 2023). Regarding distraction strategies, some early studies found that such strategies reduce participants’ memories of emotional material (Richards & Gross, 2006; Sheppes & Meiran, 2008) and promote the forgetting of negative social feedback from strangers (Xie et al., 2023). However, due to the heterogeneity of materials and tasks—for example, most of these studies used non-social, low self-related emotional images—their ecological validity is limited. This may restrict participants’ motivation for and effectiveness in emotional regulation (Nasso et al., 2020; Nelson et al., 2015). Thus, the findings from previous studies using emotional images cannot be simply extrapolated to studies on emotional regulation and memory in relation to negative social feedback from acquaintances. On the contrary, the negative emotions triggered by negative social feedback from acquaintances are highly self-relevant and significantly enhance participants’ emotional regulation, motivation, and effectiveness (Nasso et al., 2020). Therefore, this study combines the social evaluation paradigm and the emotion regulation experimental paradigm (Li et al., 2022; Nasso et al., 2020) to investigate how emotion regulation influences the emotions and memories induced by negative social feedback from acquaintances.

### 1.3. The Role of Depressive Symptoms

Another objective of this study is to investigate whether the severity of depressive symptoms influences an individual’s emotional regulation of negative social feedback. Recent studies have increasingly shown that individuals with depression exhibit deficits in processing social feedback. These deficits primarily manifest as reduced anticipation and diminished enjoyment of positive social feedback, along with a selective bias toward negative information processed primarily through bottom-up mechanisms (Y. M. Chen et al., 2021; Rappaport & Barch, 2020). Compared to healthy individuals, those with depression struggle to intentionally forget unpleasant events, exhibiting a memory bias toward negative self-related information (Rappaport & Barch, 2020). Given depressed individuals’ deficits in processing negative social information, an investigation of how emotion regulation improves mood and memory will hold significant clinical implications.

### 1.4. Aims and Hypotheses

In summary, this study aims to explore the potential benefits of emotion regulation in response to negative social feedback from acquaintances. Furthermore, it will measure participants’ depression levels and examine the relationship between depressive symptoms and the effects of emotion regulation. We anticipate that negative social feedback from acquaintances elicits stronger emotional reactions and more persistent negative memories. Emotion regulation is expected to attenuate the negative emotional responses induced by such feedback and facilitate the forgetting of negative memories. The moderating effects of emotion regulation strategies may vary across individuals with self-reported different levels of depressive symptoms.

## 2. Materials and Methods

### 2.1. Participants

A total of 73 students (51 female) from Shanxi Normal University were recruited for this study, with a mean age of 24.6 years. All participants were right-handed, had no recent illness or medication, had no history of neurologic disease or psychiatric illness, and had normal or corrected vision. The study was approved by the Ethics Committee of Shanxi Normal University. All participants signed an informed consent form before the experiment, and participants were paid accordingly at the end of the experiment. After the participants signed the informed consent form, their depression symptoms were measured by the Beck Depression Inventory second edition (BDI-II) (Beck et al., 1987), and their depression scores ranged from 0 to 30.

Ten participants’ data were excluded from the sample because their adherence to the emotion-regulation task instructions did not meet the necessary criteria. Therefore, the final sample size for data analysis included in the pre-test phase was 73 participants, and the sample size for data analysis included in the emotion-regulation and post- and delayed post-test was 63 participants.

### 2.2. Stimulus Material

According to the previous literature (Nasso et al., 2020), the positive viewing condition was only used to increase the plausibility of the experimental manipulation and test the validity of the emotional validity manipulation in the experiment (i.e., to examine whether the positive and negative social feedback elicited a corresponding emotional response). Because this paper focuses on the emotional regulation of negative social feedback, results for the positive viewing condition were not included in the statistics.

The experimental materials included ID photos of people involved in social evaluation and adjectives as social feedback. Ninety standardized ID photos—half for men and half for women—45 ID photos of acquaintances from fellow students and teachers, and 45 ID photos of strangers selected from a library of previous research materials were used in the experiment (Xie et al., 2021, 2022). Ten of the photographs were used in the practice phase of the emotion-regulation task of this experiment, and 80 photographs were used in the formal experimental task.

The other part of the material consisted of 100 personality adjectives, of which 70 were negative words and 30 positive words, selected from the Chinese emotion word system (Wang et al., 2008). Among them, 10 negative and 10 positive words were used in the preparation phase of this experiment and in the practice phase of the emotion-regulation task, leaving 60 negative and 20 positive words for the formal experimental task.

The materials used for the formal experiment described above were combined into 80 photo-word pairs of 30 acquaintance-negative evaluative pairs, 10 acquaintance-positive evaluative pairs, 30 stranger-negative evaluative pairs, and 10 stranger-positive evaluative pairs. Material attributes (gender and face attractiveness of the people in the photographs, and emotional potency and arousal of the words) were counterbalanced both within and across groups.

### 2.3. Experimental Design

**Figure 1:**
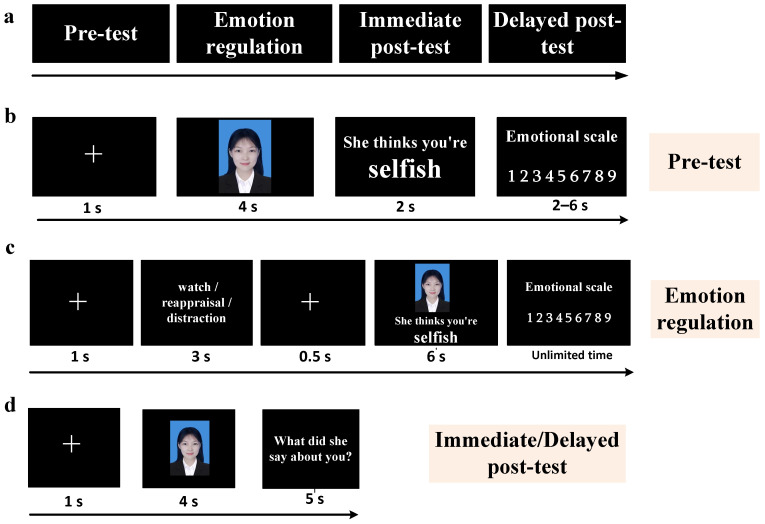
Experimental procedure. (**a**) Experimental phase and task, (**b**) flow chart of a single trial in the pre-test phase, (**c**) flow chart of a single trial for emotion regulation tasks, and (**d**) flow chart of a single trial in immediate/delayed post-test.

The experiment consisted of the following phases: preparation, pre-test, emotion regulation, immediate post-test, and delayed post-test (Figure 1a). The experimental program was written and run using E-Prime 3.0 (Psychology Software Tools, Inc., Sharpsburg, PA, USA).

**Preparation phase:** When the participants registered for the experiment, the experimenter asked them to provide a personal digital ID photo and informed them that the theme of the experiment was “first impressions evaluations” (Somerville et al., 2006). Their photos would be used as experimental materials and viewed and evaluated by their classmates, substitute teachers, and college students of the same age from other universities. Participants were informed that others would evaluate them by selecting one of two opposing personality adjectives (e.g., humble, arrogant, reliable) provided by the experimenters, according to which term best reflected the participant’s personality as assessed from the photo (Nasso et al., 2020). To enhance the credibility of the experimental procedure, participants first viewed 20 ID photos of individuals they identified as being familiar to them or strangers. Based on their first impressions of these photos, they selected one or two adjectives they believed best reflected the personality traits of the person in the photo.

Regarding the familiarity ratings of acquaintances, the participants sat in front of a computer screen and viewed 40 ID photos of classmates and teachers in random order. They rated their familiarity with the people in the photos on a 9-point scale, with 1 indicating very-low familiarity and 9 indicating very-high familiarity. Participants responded by clicking on one of the numerical options presented on the screen. There was no time limit for responding, and the next photo appeared after the response was submitted.

**Pre-test phase:** To explore the differences in emotional responses and memory strength to negative social feedback sent by acquaintances and strangers using a newly developed social feedback research paradigm in recent years. The task consisted of two blocks, each containing 80 trials presented in randomized order. The flow of each trial is shown in Figure 1b. Each trial was marked by the presentation of a “+” in the center of the screen (lasting 1 s), followed by the appearance of a person’s photograph for 4 s, and the presentation of the person’s comments about the participant (e.g., “She thinks you’re selfish,” “He thinks you are honest”). The participant is asked to rate their current emotional experience on a 9-point scale, with 1 being very negative, 5 being neutral, and 9 being very positive. Responses were made by clicking on the numerical options on the screen with the mouse. After completing the scale, the participant moved on to the next block. After completing each block, participants were given a break. Their mood ratings of negative social feedback were used to measure the level of negative mood induced by the negative social feedback. At the end of the experiment, participants verbally reported the social ratings, and the strength of the negative social feedback memory was measured by the percentage of correct recall.

**Emotion regulation phase:** The main focus was to verify the effect of emotion-regulation strategies on emotions and memories induced by the negative social feedback sent by acquaintances and strangers. Participants first completed a 15 min emotion-regulation exercise. During the exercise, the participant was introduced to the requirements and precautions of the different regulation strategies of “watching,” “reappraisal,” and “distraction” and was asked to practice each strategy until they had mastered all of them before moving on to the next stage. Specifically, for “watching,” the participants only needed to view the ID photo and feedback naturally, and no intervention was needed for any thoughts or emotional experiences that arose during the process. For “reappraisal,” participants needed to change their thoughts about the negative social feedback to reduce their negative emotional experiences—for example, imagining that “I am not like this, and if the person in the picture knew more about me, he/she would change his/her opinion about me” (Nasso et al., 2020). For “distraction,” participants were required to focus their attention on neutral thoughts unrelated to the current situation. At the end of the exercise, participants began to complete a formal emotion-regulation task. The task contained two blocks, each containing 80 trials presented in randomized order. The flow of each trial is shown in Figure 1: a 1 s gaze point was presented on the screen, followed by a 3 s emotion regulation instruction (“watch,” “reassess,” or “distract”). This was followed by a 0.5 s gaze point, followed by a photo of the person’s ID and social feedback to the participant (e.g., “He/she thinks you’re selfish,” “He/she thinks you’re honest”), with the photo and feedback presented simultaneously for 6 s. During this time, participants were required to implement the emotion-regulation instructions that had appeared earlier and perform the appropriate emotion-regulation task in response to the social feedback. Subsequently, participants rated their current emotional experience on a 9-point scale, with 1 representing very negative, 5 representing neutral, and 9 representing very positive. Responses were made by clicking on the numerical options on the screen with the mouse. After completing the rating, they then moved on to the next trial. After completing each block, participants were given a period of rest. The emotion regulation task consisted of 160 trials, as shown in Figure 1c.

Following the emotion-regulation task, participants completed a questionnaire on their adherence to the emotion-regulation task’s instructions, following the questionnaire used in the study recently published by Xie et al. (2023), which consists of four items, each of which is scored on a 5-point scale, with higher scores representing greater adherence to the instructions. If a participant did not achieve the required score (13) on the questionnaire, they followed the instruction in less than 60% of the trials; therefore, the data from this group of participants were excluded.

**Immediate post-test phase:** A total of 80 people’s ID photos were presented in randomized order, and participants were required to recall, as much as possible, what the people in the photos said about them within 5 s and report it verbally. The experimenter’s instructions to the participants were as follows: “Next, the screen will present the people’s ID photos in random order. Please recall his/her comments about you within 5 s of the appearance of the photos and answer them into the recording device. If you can remember a specific word, answer that word; if you can only remember a positive or negative comment but not the specific word, you can answer ‘positive’ or ‘negative’; if you can’t remember anything at all, answer ‘don’t remember’ (Xie et al., 2023). We will record your voice.” Five seconds later, the next picture automatically appeared, as in Figure 1d.

**Delayed post-test phase:** The day after the emotion-regulation experiment, participants returned to the laboratory to participate in a delayed post-test with the same tasks as those in the pre-test phase, as in Figure 1d.

### 2.4. Statistical Analysis

Statistical analyses of behavioral indicators were performed using GraphPad Prism 5 software. Paired-samples t-tests were used to analyze the differences in emotional responses and memory strength induced by negative social feedback sent by acquaintances and strangers; one-way repeated measures analysis of variance (ANOVA) was used to analyze the effects of the emotion-regulation strategy on mood and memory induced by negative social feedback sent by acquaintances before and after the emotion-regulation strategy, as well as differences in the adjustment of mood and memory strength by the emotion-regulation strategy for participants with self-reported different levels of depressive symptoms. The significance level was α = 0.05, and descriptive statistics are expressed as “mean ± standard deviation.”

## 3. Results

### 3.1. Emotional Scores and Memory Strength of Negative Social Feedback Sent to Different Groups of People

The one-sample t-test showed that the participants’ familiarity with classmates and teachers (8.49 ± 0.31) was significantly higher than 5 (i.e., moderate familiarity) t(72) = 18. 02, *p* < 0.001, Cohen’s *d* = 2.80.

**Figure 2:**
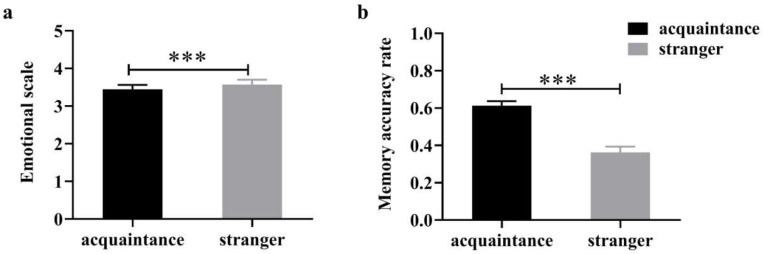
Responses induced by negative social feedback from acquaintances and strangers (**a**) emotional scale (**b**) recall accuracy rate. *** *p* < 0.001.

Paired-samples t-tests showed that participants induced significantly stronger negative emotions in response to negative social feedback sent by acquaintances compared to that sent by strangers (Figure 2a), t(72) = 1.404, *p* < 0.001, Cohen’s *d* = 3.40. Second, participants demonstrated more accurate memories of negative social feedback received from acquaintances (Figure 2b), t(72) = 8.370, *p* < 0.001, Cohen’s *d* = 2. 07. These results suggest that negative feedback given by acquaintances induces a stronger emotional response and that the associated negative emotions are remembered more deeply.

### 3.2. Effects of Emotion Regulation on Negative Social Feedback Sent by Acquaintances

**Figure 3:**
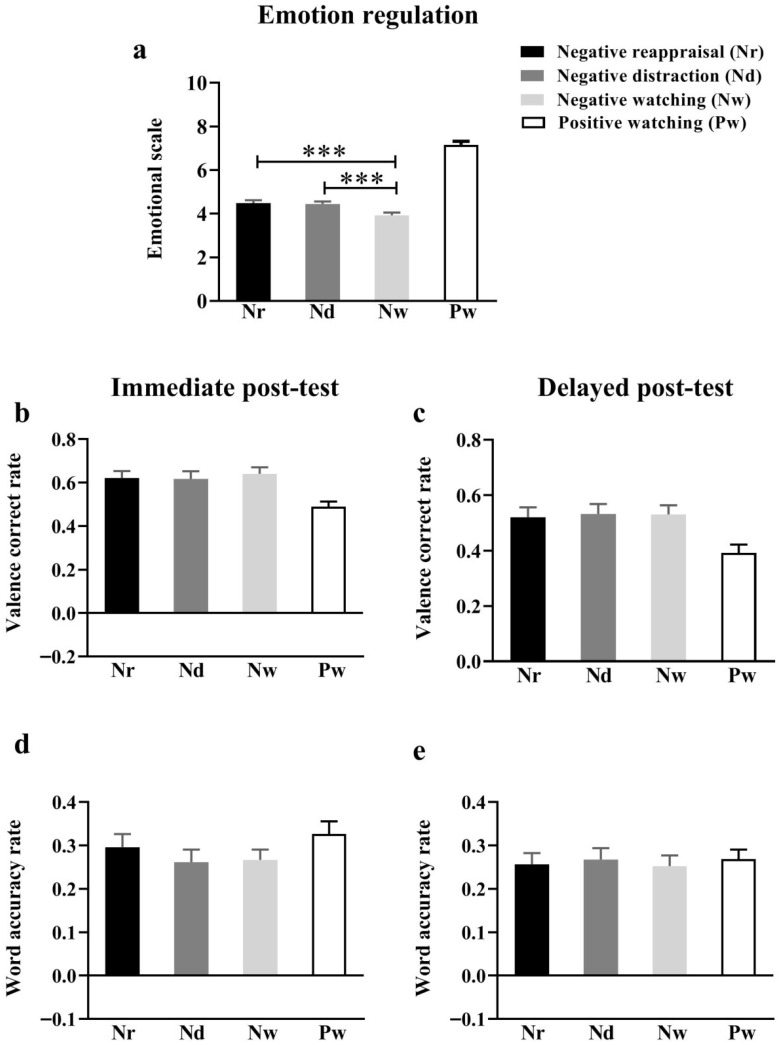
Self-reported emotional scores and recall accuracy of participants in the emotion regulation/immediate/delayed post-test. (**a**) Self-reported emotional scores in the emotion regulation, (**b**) valence correct rate in immediate post-test, (**c**) valence correct rate in delayed post-test, (**d**) word accuracy rate in immediate post-test, (**e**) W=word accuracy rate in delayed post-test. *** *p* < 0.001.

A repeated measures ANOVA for the watching, cognitive reappraisal, and distraction conditions for negative social feedback sent by acquaintances found a significant main effect of emotion regulation, *F*(2, 124) = 22.51, *p* < 0.001, $\eta_{p}^{2}$ = 0.27 (Figure 3a). Further simple effects analyses revealed that participants had increased (more positive) mood ratings in the cognitive reassessment (4.49 ± 0.02) and distraction (4.44 ± 0.02) conditions compared to the viewing condition (3.93 ± 0.02). Additionally, no significant difference was found between the ratings of the cognitive reappraisal condition and the emotion ratings of the distraction condition (*p* = 0.57); this suggests no significant difference between the effects of the cognitive reappraisal strategy and distraction on moderating negative social feedback sent by familiarizers (Figure 3a). Other than that, the regulatory efficiency of emotion regulation shows no difference between different genders (Supplementary Materials Figure S1).

The experimenter quantified the participants’ recall responses along two dimensions: (1) the accuracy rate of recalling social feedback valence (positive or negative, hereinafter referred to as valence correct rate), and (2) the accuracy rate for recalling specific social feedback words (hereinafter referred to as the word accuracy rate). In the immediate post-test phase, we found that the main effect of emotional regulation was not significant for the valence correct rate, *F*(2, 124) = 0.39, *p* = 0.68, $\eta_{p}^{2}$ = 0.01 (Figure 3b). Likewise, we found that the main effect of emotional regulation was not significant for the word accuracy rate, *F*(2, 124) = 0.70, *p* = 0.50, $\eta_{p}^{2}$ = 0.02 (Figure 3d). In the delayed post-test phase, we again found that the main effect of emotional regulation was not significant for the valence correct rate, *F*(2, 124) = 0.10, *p* = 0.90, $\eta_{p}^{2}$ = 0.002 (Figure 3c). Nor was the main effect of emotional regulation significant for the word accuracy rate, *F*(2, 124) = 0.21, *p* = 0.81, $\eta_{p}^{2}$ = 0.01 (Figure 3e). The above results suggest that neither cognitive reassessment nor distraction reduced the negative memories participants experienced after receiving negative social feedback from a familiar person.

### 3.3. The Effect of Using Emotion Regulation Strategies on Sending Negative Social Feedback from Acquaintances in Groups with Self-Reported Different Levels of Depression Scores

To further explore how self-reported depression level affects emotion-regulation processes, participants were categorized into self-reported high- and low-depressive symptom groups (10 in the self-reported high-depressive symptom group and 53 in the self-reported low-depressive symptom group) based on their scores on the Beck Depression Inventory (Beck et al., 1987), which were subjected to separate statistical tests.

**Figure 4:**
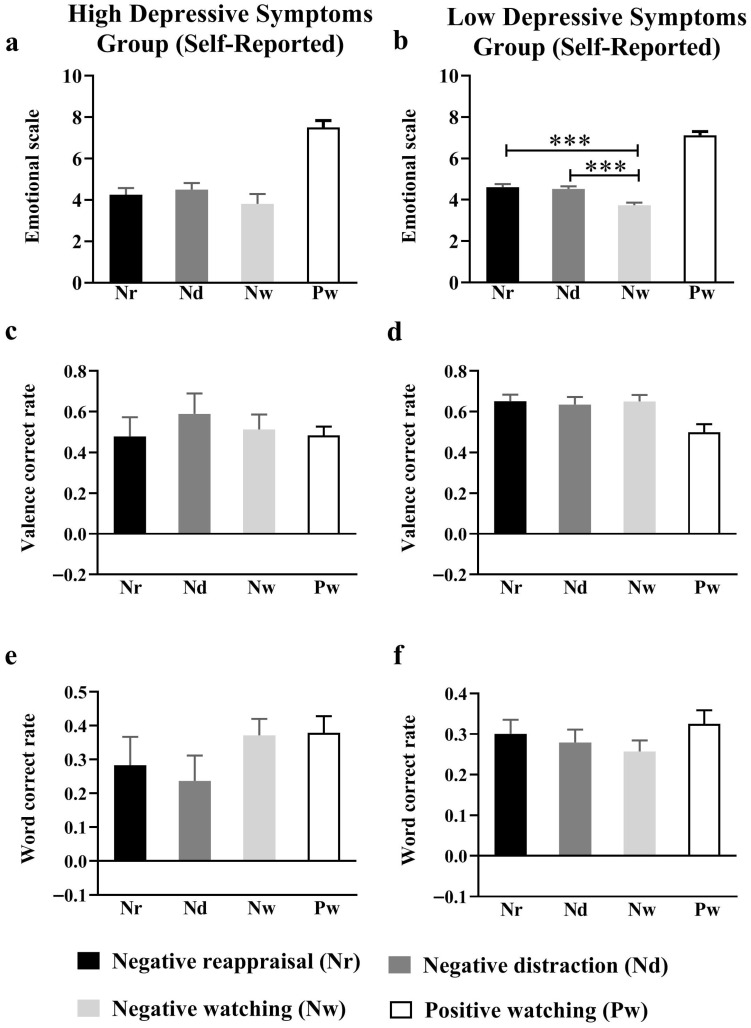
Self-reported emotional scores and recall accuracy of participants in high-/low-depression group (**a**) self-reported emotional scores in high-depression group (**b**) self-reported emotional scores in low-depression group (**c**) valence correct rate in high-depression group (**d**) valence correct rate in low-depression group (**e**) word accuracy rate in high-depression group (**f**) word accuracy rate in low-depression group. *** *p* < 0.001.

In the self-reported low-depression group, a repeated measures ANOVA for the viewing, cognitive reappraisal, and distraction conditions revealed a significant main effect of emotion regulation *F*(2, 104) = 24.90, *p* < 0.001, $\eta_{p}^{2}$ = 0.33 (Figure 4b). Further simple effects analyses revealed that participants’ mood scores increased (more positively) in the cognitive reassessment (4.62 ± 0.02, *p* < 0.001) and distraction (4.53 ± 0.02, *p* < 0.001) conditions compared to the viewing condition (3.74 ± 0.02). Additionally, no significant difference was found between scores on cognitive reappraisal and mood scores in the distraction condition (*p* = 0.32); this suggests no significant difference between the effects of cognitive reappraisal strategies and distraction on moderating negative social feedback sent by familiarizers. For participants with self-reported high-depression scores, neither emotion-regulation strategy improved participants’ mood scores, the main effect of emotional regulation was not significant *F*(2, 18) = 2.80, *p* = 0.09, $\eta_{p}^{2}$ = 0.26 (Figure 4a).

For the self-reported low-depressive symptom group, emotion regulation had no significant effect on either the valence correct nor the word accuracy rates for negative social feedback-related memories (valence correct rate: *F*(2, 104) = 0.19, *p* = 0.83, $\eta_{p}^{2}$ = 0.004; word accuracy rate: *F*(2, 104) = 0.72, *p* = 0.49, $\eta_{p}^{2}$ = 0.02) (Figure 4d,f). Nor was the main effect of emotion regulation significant in the self-reported high-depressive symptom group (valence correct rate: *F*(2, 18) = 1.23, *p* = 0.32, $\eta_{p}^{2}$ = 0.13; word accuracy rate: *F*(2, 18) = 3.40, *p* = 0.08, $\eta_{p}^{2}$ = 0.41) (Figure 4c,e).

## 4. Discussion

Research indicates that negative feedback from close social partners (such as friends and family) has a significantly greater negative impact on individuals than similar feedback from strangers (W. L. Chen & Liao, 2021). Such negative interactions within close relationships not only trigger intense mental stress and emotional distress, but they may also contribute to the development of social anxiety due to a lack of emotional support, ultimately causing sustained mental health damage (W. L. Chen & Liao, 2021). Therefore, it is important to regulate negative feedback from intimate relationships in a reasonable and effective manner. This study found that social feedback from acquaintances indeed induces stronger emotional experiences and deeper negative emotional memories. Cognitive reappraisal and distraction can reduce the emotional responses triggered by negative social feedback from acquaintances, but they do not promote the forgetting of such memories.

Further, the participants’ self-reported depression levels significantly influenced the moderating effects of different emotion regulation strategies. For participants with self-reported high-depression scores, cognitive reappraisal and distraction were ineffective in regulating the negative emotions and memories induced by negative social feedback from acquaintances. However, while reappraisal and distraction effectively regulated the negative emotions experienced by the participants with self-reported low-depression scores following negative social feedback from acquaintances, these strategies had no impact on their ability to forget the associated negative social feedback memories.

### 4.1. Negative Social Feedback Sent by Acquaintances Elicits Stronger Emotional Experiences and Is Harder to Forget

In daily life, people tend to care more about social feedback from close people or acquaintances than from strangers, which may trigger stronger emotional responses (W. L. Chen & Liao, 2021). This is consistent with our expectations. Individuals usually have closer emotional connections and higher levels of trust with people they know well. Consequently, evaluations of these closely connected people are more likely to affect their inner sense of self-worth. Therefore, social feedback from people they know often has a deeper and more lasting impact on individuals (S. Chen et al., 2006). Specifically, individuals feel more pleasant with positive social feedback sent by acquaintances and sadder with negative social feedback sent by acquaintances (Xie et al., 2022). Evidence from fMRI further supports this view. Studies have shown that in facial recognition memory tests, recognition of unfamiliar faces activates only the hippocampus, while recognition of familiar faces activates both the hippocampus and the amygdala (Somerville et al., 2006). This further suggests that social feedback from acquaintances induces stronger emotional experiences than that from strangers. Moreover, emotional valence influences memory strength.

### 4.2. Effects of Emotion Regulation Strategies on Emotions and Memories Elicited by Negative Social Feedback from Acquaintances

The present study used the highly self-relevant social feedback material from acquaintances to examine how different emotion-regulation strategies affect the forgetting of negative social feedback information. We found that both reappraisal and distraction increased participants’ emotion ratings of negative social feedback compared to passive viewing, but they had no effect on negative social feedback memory. Previous studies have shown that cognitive reappraisal and distraction can both effectively reduce negative emotions (Nelson et al., 2015; Craik, 2014; Sheppes & Meiran, 2008). Furthermore, Xie et al. also found that cognitive reappraisal and distraction can effectively reduce the negative emotions triggered by negative social feedback from strangers (Xie et al., 2023). Our findings suggest that reappraisal and distraction are effective in reducing the negative effects of negative social feedback on mood and improving mood scores, and no difference exists in the effectiveness of reappraisal and distraction in reducing the experience of negative mood. This is consistent with the results of previous studies; it reaffirms that the two emotion-regulation strategies of reappraisal and distraction are useful in regulating emotions and that this regulation is as effective for acquaintances as for strangers.

Notably, previous studies have found that cognitive reappraisal and distraction strategies reduce participants’ memories of emotional material (Richards & Gross, 2006; Sheppes & Meiran, 2008; Xie et al., 2023). On the other hand, our study found that emotional regulation strategies do not facilitate the forgetting of memories related to negative social feedback from acquaintances. There are several possible explanations for this result. First, considering the heterogeneity of the materials and tasks, emotional regulation strategies may be ineffective in reducing negative emotional memories. Most studies use non-social, low self-related emotional images, such as frightening snakes and beasts, or devastating natural disasters. These emotional stimuli have certain limitations in terms of ecological validity, which may restrict participants’ motivation for and effectiveness in emotional regulation (Nasso et al., 2020; Nelson et al., 2015). For example, negative social feedback from strangers may be less emotionally impactful, resulting in weaker emotional responses and memory formation. This, in turn, may lead to inconsistent outcomes when using emotional regulation strategies. This also suggests the need to further assess the applicability of cognitive reappraisal, distraction, and other emotional regulation strategies in different contexts and with different targets.

Secondly, there is currently no consensus on how emotion regulation strategies influence memory. For example, some studies suggest that because cognitive reappraisal involves the deep processing of emotional material, it enhances memory encoding and subsequent memory performance (Davis & Levine, 2013; Hayes et al., 2010; Richards et al., 2003), Other studies, however, propose that since cognitive reappraisal is an antecedent-focused emotion regulation strategy (i.e., participants initiate cognitive transformation before the specific emotional material appears; Gross, 1998), it does not affect the encoding and memory of emotional material (Egloff et al., 2006; Richards & Gross, 2000; Sheppes & Meiran, 2008). Still, others suggest that participants reduce the self-relevance of current negative feedback by imagining that the evaluator does not understand them, thereby promoting the forgetting of negative social feedback (Xie et al., 2023). However, from the standpoint of neural regulation, based on the above evidence, cognitive reappraisal relies on regulation from the vlPFC to the amygdala, and distraction strategies similarly depend on the prefrontal cortex’s allocation of cognitive resources. According to Baumeister and Leary (1995), humans have a strong need for belonging. When negative feedback from closely related people triggers social rejection, it leads to increased cortisol release, which weakens prefrontal cortex function (Liston et al., 2009). Therefore, we hypothesize that when receiving negative social feedback from acquaintances, the functioning of the prefrontal cortex, which is critical for cognitive reappraisal and distraction, is inhibited, leading to excessive and prolonged amygdala activation. This, in turn, promotes hippocampal overactivation, which is the core brain region for episodic memory. This facilitates the retrieval of negative information (Disner et al., 2011), thereby enhancing negative emotional memory. Another possibility is that cognitive reappraisal and distraction induce memory reconsolidation through memory re-retrieval, thereby strengthening negative emotional memories. Of course, more experimental evidence is needed to verify this hypothesis.

Thirdly, individuals with different attachment styles exhibit varying degrees of effectiveness when employing emotion regulation strategies. For example, anxious and avoidant individuals do not experience enhanced forgetting of negative emotional memories following the use of emotion regulation strategies (Fraley et al., 2000). Since this study did not measure participants’ attachment styles, this hypothesis requires further exploration.

### 4.3. Emotion Regulation Strategies Differ in Their Effectiveness in Moderating Negative Social Feedback in Self-Reported High- and Low-Depressive Symptoms Groups

Further, considering that individuals with depression, a common psychopathological disorder, tend to perform poorly in interpersonal interactions and may suffer from impaired emotion regulation, the present study also explored the differences in the moderating effects of emotion-regulation strategies when individuals with different self-reported levels of depression receive negative social feedback. We found that for participants with self-reported high-levels of depressive symptoms, both the reappraisal and distraction conditions were ineffective in regulating negative emotions and memories induced by negative social feedback sent by acquaintances. Conversely, for those with self-reported low-depressive symptoms, reappraisal and distraction were effective in regulating negative emotions induced by negative social feedback sent by acquaintances but had no effect on forgetting memories of negative social feedback. Since cognitive reappraisal involves the interaction between cognitive control and emotional processing, cognitive abilities and emotional reactivity may influence cognitive reappraisal ability. Individuals with depressive disorders typically exhibit poorer cognitive control abilities (Dotson et al., 2020) and distinct negative emotional reactivity (Bylsma, 2021; Kivity & Huppert, 2016), which might explain why this study’s self-reported high-depressive symptom group exhibited poorer cognitive reappraisal ability (Zilverstand et al., 2017).

First, there are significant differences in emotional reactivity between individuals with depressive disorders and healthy individuals. Specifically, the theory of emotional insensitivity posits that a core feature of depression is a reduction in both positive and negative emotional responses (Bylsma, 2021; Cui et al., 2024). Therefore, this study’s self-reported high-depressive symptoms group may also be constrained by the floor effect, which results in significantly less cognitive reappraisal of negative stimuli compared to the self-reported low-depressive symptoms group (Cui et al., 2024). Second, individuals with depression may exhibit other cognitive reappraisal deficits. For example, functional connectivity, including connectivity between the frontal lobe and amygdala regions, in performing cognitive reappraisal tasks, differs between individuals with depression and healthy individuals (Perlman et al., 2012; Platt et al., 2015; Cui et al., 2024).

Furthermore, such deficits may also manifest in the sustainability of reappraisal ability (Cui et al., 2024). At the behavioral level, Erk et al. (2010) found that both individuals with depression and healthy individuals can alter their emotions through reappraisal. However, the reappraisal effect can last up to 15 min in healthy individuals, while the reappraisal effect in individuals with depression does not persist. At the neuroimaging level, although depressed patients and healthy individuals showed no differences in brain activation during the first half of the reappraisal task, the intensity of brain activation in depressed patients significantly decreased during the latter half of the task (Heller et al., 2009). In summary, the high-depression group experienced a lesser reduction in negative emotions than the low-depression group, indicating that their cognitive reappraisal abilities were deficient. Effective distraction strategies actively shift attention away from negative information toward neutral/positive stimuli. However, individuals with self-reported high-depressive symptoms, might, due to their inhibitory dysfunction and impaired prefrontal cortex (PFC) function (particularly in the dorsolateral prefrontal cortex, dlPFC), experience reduced attention control capabilities. Thus, it is not easy to effectively execute attention shifting (Y. M. Chen et al., 2021). As a result, participants with self-reported high-depressive symptoms fail to regulate negative emotions using distraction strategies.

Another possible reason for the present results is the reality of emotional overgeneralization. Self-reported depressive symptoms individuals may exhibit attentional bias and excessive rumination toward high self-related negative evaluations from acquaintances, which may generalize to overall doubts of their self-worth. This prevents distraction from blocking deep-seated self-negativity, thereby failing to alleviate negative emotions. The failure of emotion regulation strategies to enhance forgetting negative memories in both self-reported high- and low-depressive symptom groups has been addressed in Section 4.2 and will not be reiterated here.

The present study examines the phenomenon that negative social feedback from acquaintances activates the social pain network and triggers stronger emotional responses. By investigating the moderating effects of cognitive reappraisal and distraction strategies in different social relationship contexts, it not only deepens our understanding of emotional regulation mechanisms in intimate relationships, but also reveals the differential effects of emotional regulation strategies on emotional experiences and emotional memories across different contexts. Moreover, it also provides valuable clinical insights for future research on how to more effectively alleviate social pain in individuals with high-depressive symptoms. On a practical level, these findings suggest that enhancing individual emotion regulation capacity (Emily et al., 2021)—particularly through interventions that are longer than one session and designed for a broader age range—may help mitigate the negative effects of adverse social feedback from acquaintances.

This study has five main shortcomings. First, the sample size of the high-depression group was small and the high- and-low depression subgroups in this study relied on subjective scale report scores rather than clinical diagnoses of depression. Therefore, future research is needed to investigate whether the results of this study can be extrapolated to patients with clinically diagnosed depression. Second, given the limited number of male participants and the substantial gender imbalance in the current sample, coupled with the established gender differences in emotion regulation capabilities, future studies should aim to balance sex ratios and incorporate gender as an analytical variable. Third, this study has an absence of self-reported measures for assessing emotion regulation strategies and positive/negative affect indicators, which are crucial variables for understanding the psychological mechanisms underlying the observed effects. Fourth, the extended and multi-phase nature of the experimental procedure may have induced participant fatigue, potentially contributing to attentional lapses or reduced motivation to fully adhere to task instructions. Future studies may consider incorporating attention checks, dividing lengthy tasks into shorter sessions, or using more immersive paradigms to sustain engagement and reduce fatigue-related attrition. Fifth, as mentioned earlier, the present study found that cognitive reappraisal and distraction did not reduce individuals’ memory for negative social feedback sent by acquaintances, which is inconsistent with the results of previous studies. Future research is needed to further validate the role of emotion-regulation strategies on the psychological and neural mechanisms of emotional-memory forgetting behavior and to expand the ways to promote the forgetting of negative social feedback.

## Data Availability

The datasets generated and analyzed in the current study are available from the corresponding (first) author upon reasonable request.

## Supplementary Materials

The following supporting information can be downloaded at: https://www.mdpi.com/article/10.3390/bs15091294/s1, Figure S1: Self-reported emotional scores in the emotion regulation in male and female.
