# Correction: Liu et al. (2025). Emotion Regulation Modulates Affective Responses Without Altering Memory Traces: A Study of Negative Social Feedback from Acquaintances. *Behavioral Sciences*, *15*(9), 1294

**DOI:** 10.3390/bs15111470

**Published:** 2025-10-29

**Authors:** Peng Liu, Xin Cheng, Mengyao Fan, Zhichao Huang, Chao Zhang

**Affiliations:** School of Psychology, Shanxi Normal University, Taiyuan 030031, China

**Text Correction**

There was an error in the original publication (Liu et al., 2025). The **mistake** was in the Results section, “3.1. Emotional Scores and Memory Strength of Negative Social Feedback Sent to Different Groups of People”.

A correction has been made to the Results, 3.1. Emotional Scores and Memory Strength of Negative Social Feedback Sent to Different Groups of People, Paragraphs 1 and 2:

The one-sample t-test showed that the participants’ familiarity with classmates and teachers (6.70 ± 1.23) was significantly higher than 5 (i.e., moderate familiarity): t(72) = 11.78, *p* < 0.001, Cohen’s *d* = 1.38.

Paired-samples t-tests showed that participants induced significantly stronger negative emotions in response to negative social feedback sent by acquaintances compared to that sent by strangers (Figure 2a): t(72) = −5.102, *p* < 0.001, Cohen’s *d* = 0.60. Second, participants demonstrated more accurate memories of negative social feedback received from acquaintances (Figure 2b): t(72) = 8.370, *p* < 0.001, Cohen’s *d* = 0.98. These results suggest that negative feedback given by acquaintances induces a stronger emotional response and that the associated negative emotions are remembered more deeply.

**Error in Figure**

In the original publication, there was a mistake in “**Figure 2**” as published. “**Figure 2. Responses induced by negative social feedback from acquaintances and strangers**”.

The corrected “**Figure 2**” appears below.

**Figure 2:**
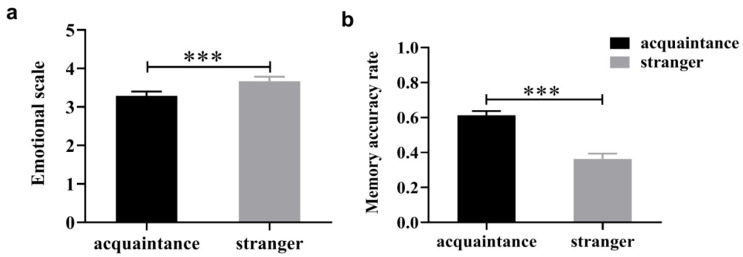
Responses induced by negative social feedback from acquaintances and strangers: (**a**) emotional scale; (**b**) recall accuracy rate. *** *p* < 0.001.

The authors state that the scientific conclusions are unaffected. This correction was approved by the Academic Editor. The original publication has also been updated.
