# Supplementary material for: Emotion Regulation Modulates Affective Responses Without Altering Memory Traces: A Study of Negative Social Feedback from Acquaintances
Source: Behav Sci (Basel). 2025 Sep 22;15(9):1294. doi: 10.3390/bs15091294 (PMC12467585; doi:10.3390/bs15091294)
Supplement: Supplementary file 1 [file behavsci-15-01294-s001.zip › behavsci-3838696-supplementary.pdf]

A repeated measures ANOVA indicated that a significant main effect of emotion regulation in male $F(2, 20)=8.231$, $p =0.0025$, $\eta 2\ p=0.18$ (figure S1A). Similarly, a significant main effect of emotion regulation was also found in female $F(2, 102)=17.09$, $p <0.0001$, $\eta 2\ p=0.23$ (figure S1B). The results indicated that the regulatory efficiency of emotion regulation shows no difference between different genders.

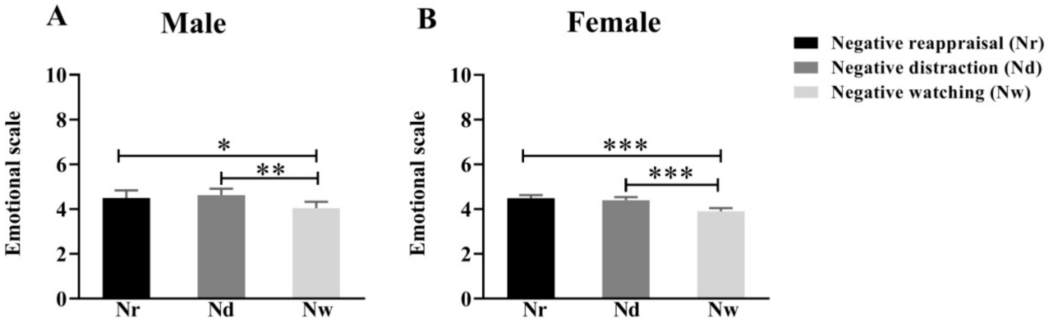

Figure S1. Self-reported emotional scores in the emotion regulation in male and female. *** P < 0.001
